# Iodine Activation from Iodate Reduction in Aqueous Films via Photocatalyzed and Dark Reactions

**DOI:** 10.1021/acsearthspacechem.4c00224

**Published:** 2024-12-03

**Authors:** Mago Reza, Lucia Iezzi, Henning Finkenzeller, Antoine Roose, Markus Ammann, Rainer Volkamer

**Affiliations:** † Department of Chemistry, University of Colorado Boulder Boulder, Colorado 80309, United States; ‡ Cooperative Institute for Research in Environmental Sciences (CIRES), University of Colorado Boulder Boulder, Colorado 80309, United States; § PSI Center for Energy and Environmental Sciences, Villigen 5232, Switzerland

**Keywords:** iodine recycling, iodate reduction, dark reactions, photocatalyzed reactions, photochemistry

## Abstract

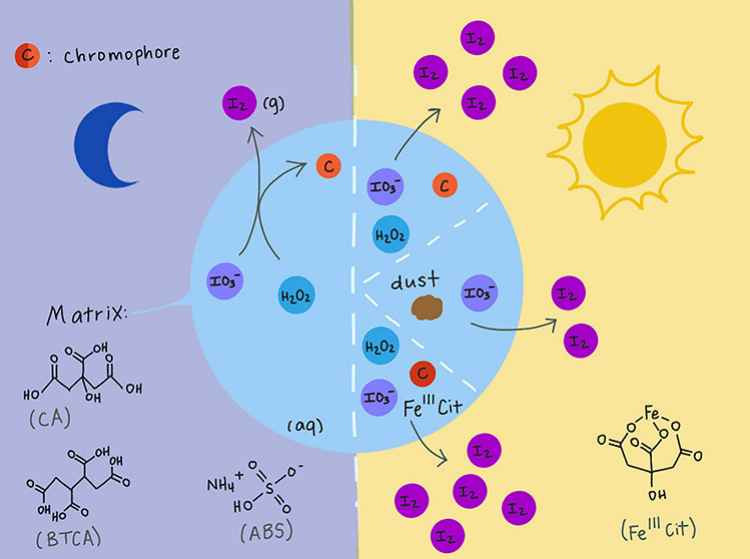

Iodine in the atmosphere destroys ozone and can nucleate particles by formation of iodic acid, HIO_3_. Recent field observations suggest iodate recycles from particles sustaining significant gas-phase IO radical concentrations (0.06 pptv) in aged stratospheric air, and in elevated dust plumes. However, laboratory evidence for iodine activation from aerosols is currently missing. Here, a series of coated-wall flow tube (CWFT) experiments test for iodine release from thin aqueous films containing iodate. Photocatalyzed reactions were studied using iron(III) citrate (Fe–Cit), Arizona Test Dust (ATD), and Fe_2_O_3_, along with the dark reaction of iodate with H_2_O_2_ at 90% RH and 293 K. Fresh films were separately irradiated with visible and UV-A light, and the efficient release of molecular iodine, I_2_, was observed from all irradiated films containing photocatalysts. For films with Fe–Cit, visible light reduced larger amounts of iodate than UV-A light, activating ∼40% of iodate as I_2_. The formation of oxygenated volatile organic compounds (OVOC) and iodinated OVOC was also observed. Dark exposure of films to H_2_O_2_ led to I_2_ release in smaller amounts than suggested by Bray–Liebhafsky kinetics, consistent with H_2_O_2_ salting-out in the films, or possibly other reasons. Photochemical activation is enhanced by dust proxies in the film, and by aging the film with H_2_O_2_ in the dark prior to irradiation. These findings help explain recent field observations of elevated IO radical concentrations in lofted dust layers, and warrant the inclusion of photocatalyzed iodate reduction in atmospheric models.

## 1 Introduction

Iodine emissions from the oceans to the atmosphere have increased by a factor of 3 in recent decades due to tropospheric ozone pollution.^1^ Atmospheric iodine destroys ozone^2−5^ and efficiently forms particles from the nucleation of iodine oxoacids.^6,7^ On a per atom basis, iodine destroys O_3_ more efficiently than bromine by a factor ∼6, and chlorine by 3 orders of magnitude.^8^ The nucleation rate of iodic acid, HIO_3_, is faster than that of sulfuric acid-ammonia by a factor of ∼21–33.^6^ The formation mechanism of HIO_3_ bridges gas-phase iodine to new particle formation and indicates three O_3_ molecules are consumed to make one HIO_3_ molecule.^9^ Iodate, IO_3_^–^, is the most thermodynamically stable form of iodine,^10,11^ multiphase chemistry models such as CAPRAM halogen module 3.0 therefore currently accumulate aqueous iodine as iodate in particles.^12^ However, the coexistence of gas-phase IO radicals with particulate iodine (IO_3_^–^, I^–^) in aged stratospheric air suggests a recycling mechanism is at play that prevents iodate accumulation as a terminal product.^8,13^ In addition, recent aircraft observations of enhanced IO radicals in lofted desert dust layers have found dust is a source of gas-phase iodine to the free-troposphere.^14^ While recent work has postulated the photolysis of iodate into gas phase iodine occurring at UV wavelengths, λ < 300 nm,^13^ such high energy photons may be relevant in the middle stratosphere, but do not reach the troposphere. The molecular speciation and mechanism of iodine release from particulate iodate or dust is currently not well understood. Notably, the molecular form in which iodine recycles back to the gas phase determines the efficiency of multiphase iodate recycling as a presently unrecognized pathway for O_3_ destruction. Understanding how particulate iodate is activated back to the gas phase is necessary for better constraining the iodine partitioning, lifetime, catalytic aerosol formation, and O_3_ destruction from iodine.

Previous work finds a rich iodine multiphase chemistry that includes pathways for iodate reduction. Dark recycling reactions of iodate by nitrite,^15^ hydrogen peroxide, H_2_O_2_,^16,17^ Fe-organic matter associations,^18^ and alcohols,^19,20^ along with photosensitized reduction of iodate,^21^ and photolysis of frozen iodate salts^22^ under near UV–visible light have been studied in nanoparticles, aqueous bulk or frozen solutions. Here, we study concentrated aqueous films containing iodate in concentrations that relatively resemble stratospheric aerosols, but at warm temperatures. The photocatalyzed and dark reduction of iodate is investigated in inorganic and organic films serving as aerosol proxies through a series of coated-wall flow tube (CWFT) experiments.

We probe photocatalyzed reactions with iron, one of the most abundant transition metals found in the Earth, with iron bearing minerals representing the third most abundant class of metal oxides by mass in the Earth’s crust.^23^ Iron-containing aerosols originate mostly from mineral dust transported into the atmosphere by wind or by anthropogenic activities. The abundance of iron in tropospheric aerosol is highly variable and dependent on location and amount of aerosol liquid water (ALW). Fe concentrations have been estimated to range from 5 to 24 mM (considering a typical ALW content of 6 × 10^–8^ L m^–3^ of air, and iron concentrations in fine particles of 331–1640 ng m^–3^ from China).^23^ We chose iron(III) citrate (Fe–Cit) as a proxy for Fe(III) carboxylate complexes found in the atmosphere, as it is a well-known photoactive compound.^24−27^ A number of organic and inorganic matrices are chosen to contrast with the photocatalyzed Fe–Cit system: the citric acid (CA) matrix (in absence of iron) is an established proxy for atmospheric oxygenated organics, with its hygroscopic properties being well characterized,^28,29^ making it a natural choice as an organic matrix. In addition, we chose 1,2,3,4-butanetetracarboxylic acid (BTCA) as a second organic matrix that lacks the hydroxyl group-oxidation site found in CA and is less prone to oxidation, avoiding potential chromophore production upon irradiation. Ammonium bisulfate (ABS) was chosen for an inorganic matrix without any known chromophores within the actinic region of our setup, and given that sulfate is a dominant component of inorganic aerosol, due to the release of SO_2_ from anthropogenic activities.^30^ Arizona Test Dust (ATD) and iron(III) oxide (hematite, Fe_2_O_3_) were chosen as dust proxies motivated by field evidence for iodine recycling on dust aerosols.^14^

Molecular iodine, I_2_, evaporated from films, was detected by Cavity Enhanced Differential Optical Absorption Spectroscopy, CE-DOAS, and I-CIMS measurements provided a look at the chemical speciation of an irradiated film containing photoactive Fe–Cit. The experimental setup, conditions, instrumentation, methods, and calculations used are discussed in Section 2. Section 3 presents results and discusses atmospheric implications for (1) photochemical experiments of films with and without photoactive Fe–Cit, under UV-A and VIS light; (2) photochemical dust experiments under VIS light; (3) dark experiments of iodate and H_2_O_2_; where H_2_O_2_ serves as a reducing agent according to part of the Bray–Liebhafsky (BL) mechanism; (4) dark experiments followed by visible irradiation, where production of a chromophore arises from the aging of films with H_2_O_2_; and (5) chemical speciation from a photochemical experiment under VIS light, with Fe–Cit part of the film. Finally, Section 4 concludes on the major findings and suggests areas for further laboratory experimentation and model development.

## 2 Methods

A series of coated-wall flow tube (CWFT) experiments were conducted to investigate the release of gas phase I_2_ from photochemical experiments, where an aqueous film containing iodate was irradiated in either absence or presence of a photoactive compound under visible and UV-A light, separately (Section 2.2.1), and exposed to H_2_O_2_ in the dark (Section 2.2.2).

### 2.1 Coated-Wall Flow Tube Set up

**Figure 1:**
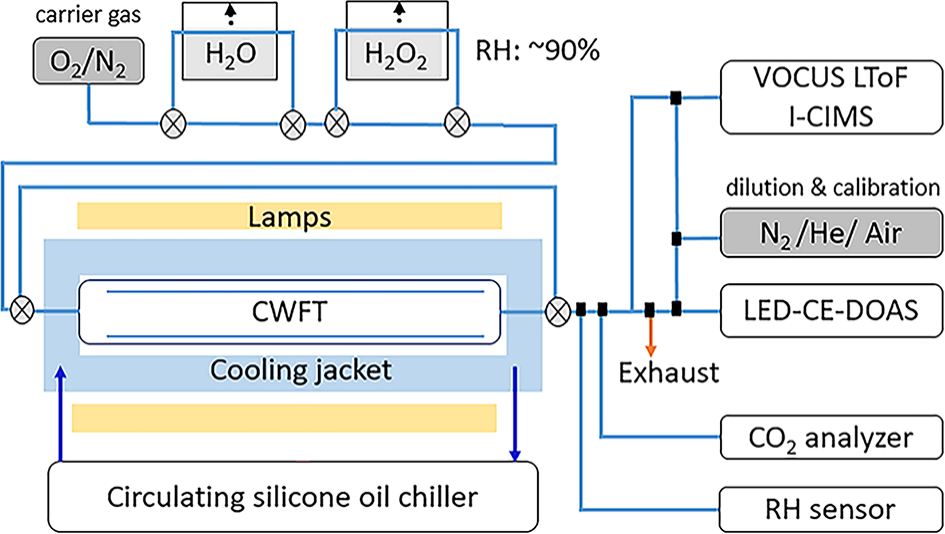
Flow-tube reactor set up.

**Figure 2:**
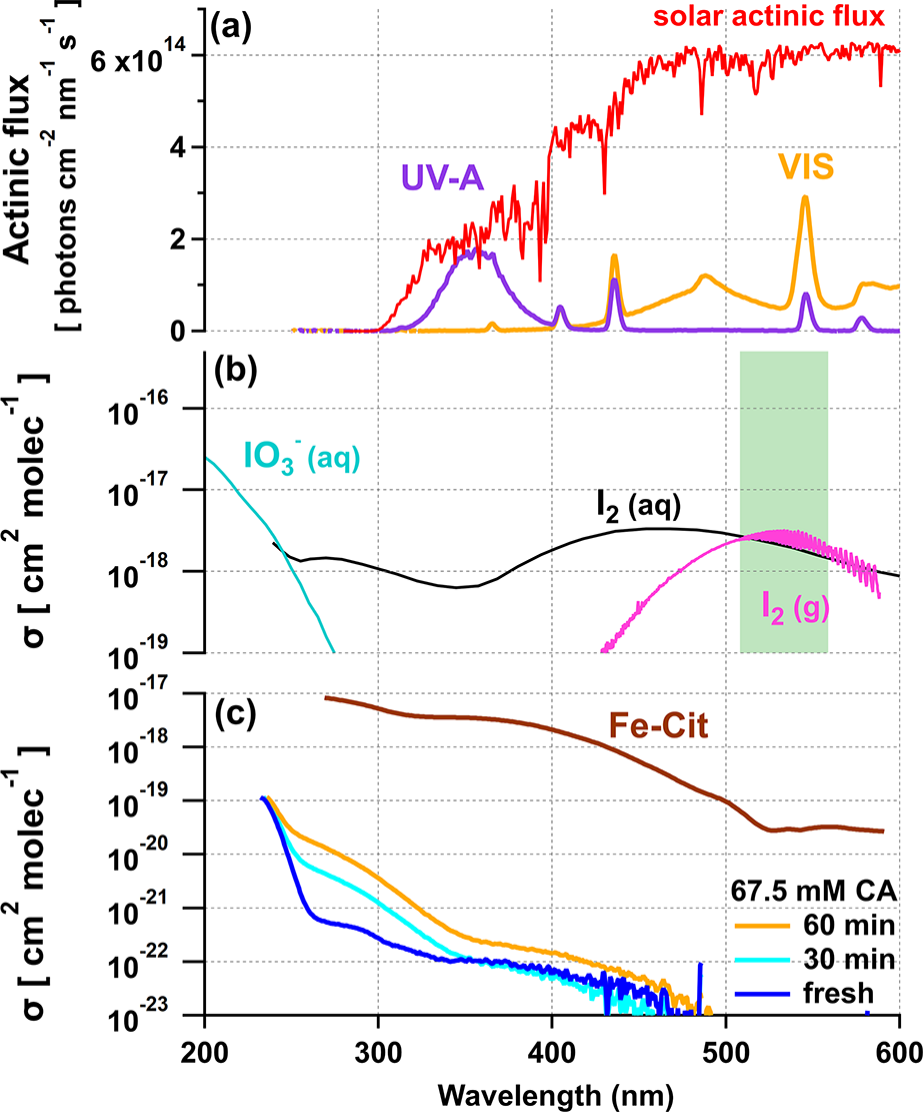
Actinic flux of lamps (UV-A and VIS) and solar spectrum (NCAR TUV calculator, overhead sun at sea surface, equator, for 30 June, 23.19 degree Solar Zenith Angle) (a). Absorption cross-sections of iodine species: ammonium iodate solution,^22^ aqueous^44^ and gas-phase I_2_^45^ (b). The absorption cross-section of a CA solution (67.5 mM) is shown for the freshly prepared solution (blue), after 30 min of measurement (light blue), and after 60 min of measurement (yellow) (c).

The experimental set up is shown in Figure 1. The CWFT reactor consists of a glass tube (Duran glass); 0.54 cm inner radius, 36.0 cm length, 122.71 cm^2^ inner surface) housed in a cooling jacket to control the temperature at 293.0 K. Humidified N_2_ and O_2_ were mixed and used as carrier gas at a total flow rate of 1.4 lpm (N_2_:O_2_ ratio of 2.5:1) or 1.7 lpm (1.43:1) set by mass flow controllers, resulting in a residence time in the reactor tube of 1.2–1.4 s. Relative humidity was controlled at 90% RH ± 3.0% by flowing the carrier gas through a temperature controlled bubbler (containing water or ∼0.1 M H_2_O_2_) prior to introducing it into the CWFT reactor. The cooling jacket was surrounded by a photoreactor consisting of 3 UV-A lamps (Philips Cleo Effect 20 W: 315–420 nm) and 4 visible lamps (Osram Warm White 15W). The actinic flux of the lamps used in this flow tube reactor are shown in Figure 2.

### 2.2 Film Preparation and Conditions

Experimental conditions are summarized in Table 1. A glass tube was coated with a solution composed of sodium iodate, NaIO_3_, and a matrix at a concentration ratio of 1:100. The concentration of iodate in the film was ∼30 mM, within a factor of 2.5–4.5 of the estimated lower and upper-limit for expected concentrations of iodate in atmospheric aerosols in the upper troposphere-lower stratosphere (UTLS), ∼75–135 mM (see Supporting Information text for details).^8^ One of the three following matrices were used for each film coating: (1) ammonium bisulfate (ABS), 1,2,3,4-butanetetracarboxylic acid (BTCA); (2) citric acid (CA); or (3) CA and iron(III) citrate (Fe–Cit).

**Table 1:** All Experiments Were Conducted at a Temperature of 20 °C and RH of ∼90%^d^

film matrix	light	H_2_O_2_ in CWFT (ppmv)	film vol. (mm^3^)	film conc. (M) IO_3_^–^/matrix	time (h)	max Δ*I*_2_ (ppbv)	IO_3_^–^_consumed_ (%)
ABS	VIS	0	28.3	0.030/2.83	0.34	0.03	0.0024
ABS	UVA	0	28.3	0.030/2.83	0.91–15.70	0.15	0.072–0.62
ABS/ATD	VIS	0	28.5	0.028/2.83/-	1.04–65.94	0.20	0.052–1.6
ABS/Fe_2_O_3_	VIS	0	28.3	0.029/2.83/-	1.00	0.10	0.055
ABS	dark, VIS	0.65	28.4	0.031/2.83	28.50^a^/0.30^b^	0.37/0.01^c^	0.36
BTCA	VIS	0	31.2	0.026/2.57	0.42	0.030	0.0020
BTCA	UVA	0	31.2	0.027/2.57	1.36	3.00	1.2
BTCA/ATD	VIS	0	31.1	0.026/2.57/-	0.81	0.04	0.0012
BTCA/Fe_2_O_3_	VIS	0	31.2	0.026/2.57/-	0.39	0.06	0.0017
BTCA	dark, VIS	0.65	31.2	0.027/2.57	2.85^a^/0.18^b^	0.20/0.04^c^	0.30
CA	VIS	0	26.1	0.036/3.07	0.81	0.04	0.0036
CA	UVA	0	26.1	0.034/3.07	29.87	2.25	27
CA/Fe–Cit	VIS	0	28.5	0.030/2.82/0.290	18.05	41.19	40
CA/Fe–Cit	UVA	0	28.5	0.030/2.82/0.287	2.44–17.17	176.44–235.83	21–32
CA	dark, VIS	0.65	26.1	0.039/3.07	16.32^a^/3.41^b^	6.77/0.01^c^	13
CA	dark, VIS	0.65	26.1	0.036/3.07	2.20^a^/62.02^b^	2.51/0.02^c^	64
CA	dark, VIS	0.65	26.3	0.030/3.07	16.35^a^/1.45^b^	1.70/0.01^c^	2.3
CA	dark	0.65	26.0	0.15/3.07	15.27^a^	0.01^c^	0.018
CA	dark	3.3	26.1	0.15/3.07	67.17^a^	0.08^c^	37
CA	dark	3.3	26.1	0.030/3.07	18.55^a^	0.16^c^	3.1

a Time that the film was exposed to H_2_O_2_ in the dark.

b Time that the film was irradiated with visible light after dark aging with H_2_O_2_.

c Average Δ*I*_2_ in the dark, see details in the text.

d The residence time in the reactor was 1.2–1.4 s. Each line shows the average or range of the result from 2 to 4 repeat experiments. Abbreviations: ABS (ammonium bisulfate), BTCA (1,2,3,4-butanetetracarboxylic acid), CA (citric acid), Fe–Cit (iron(III) citrate), ATD (Arizona test dust).

All solutions were prepared using ultrapure water (resistivity 18 MΩ cm). The iodate solution was prepared by making a 50 mL solution of 0.001 M NaIO_3_; 9.9 mg of NaIO_3_ were weighed and dissolved in ultrapure water. Then, 10 mL of the NaIO_3_ solution was used to dissolve the appropriate amount of the matrix compound (ABS, BTCA, or CA) to end up with 0.1 M matrix solution. If Fe–Cit was added to the iodate/CA solution, it was prepared at a concentration ratio of 1:10:100 (NaIO_3_: Fe–Cit: CA) and spun for ∼1 h. Solutions containing a dust proxy (ATD or Fe_2_O_3_) were sonicated for ∼1 h and spun ∼1 h to aid in the formation of a suspension. ATD is composed of silica and metal oxides, with iron constituting 4–7% of the mixture of powdered minerals containing a mixture of various iron oxides.^31,32^ ATD was added at a mass ratio of 1:10 (ATD: matrix). Fe_2_O_3_ was added at a concentration ratio of 1:10:100 (NaIO_3_: Fe_2_O_3_: matrix).

For dark experiments, 100 mL of 0.098 M H_2_O_2_ solution was prepared by mixing 1 mL of 30% H_2_O_2_ (9.8 M) with ultrapure water. This was then transferred into a bubbler to provide H_2_O_2_ gas into the carrier gas flow. A fritted bubbler served to burst any emerging bubbles, preventing microdroplets from entering the gas flow. The H_2_O_2_ concentration of ∼0.1 M in the bubbler is on the same order of magnitude as previous works studying the reduction of iodate by H_2_O_2_. The addition of H_2_O_2_ in the gas-phase (rather than the film) provides for experimental control to study the effect of H_2_O_2_ under well-defined starting conditions, and avoids evaporative or reactive losses within/from the film during experiment preparation; the equivalent gas-phase concentration of 0.65 ppmv in the carrier gas flow (see Supporting Information for calculation details) is about 2 orders of magnitude larger than gas-phase H_2_O_2_ observed in the lower troposphere, where 2–3 ppbv H_2_O_2_ are typically observed in remote air over the tropical Atlantic Ocean,^33^ and can be in excess of 10 ppbv H_2_O_2_ in polluted regions.^34^ The concentration of H_2_O_2_ in the aqueous film is estimated to be ∼0.082 M based on expected Henry’s law partitioning (see Supporting Information for calculation details). While this is about a factor of 16–30 higher than the estimated concentration of H_2_O_2_ in the aerosol-phase in the UT (0.0027–0.005 M, see Supporting Information text for details),^35^ the residence time of air in the UT (∼10 days) is 6 orders of magnitude longer than in our setup,^14^ allowing reactions in atmospheric particles to proceed over longer time scales.

The experimental procedure was adjusted to minimize I_2_ backgrounds. This was accomplished by optimizing the cleaning of glassware; the glass tubes were cleaned with absolute ethanol, acetone, hydrochloric acid, 1.0 M HCl, and sodium hydroxide, 1 M NaOH. It was then rinsed with ultrapure water and left to completely dry. The lines were flushed with N_2_, the cooling jacket was rinsed with ultrapure water, and the empty jacket was irradiated with visible and UV-A light, separately, to confirm the I_2_ background was negligible; only then, the cleaned-uncoated tube was introduced and irradiated to test the I_2_ background was still negligible, prior to proceeding with coating the tube. To create the film coatings, 800 μL of solution was pipetted into the glass tube. The coating process involved rotating and tilting the glass tube while humidified air passed through it. This was done until a thin homogeneous film was visible on the inside walls of the tube. The homogeneity of the film was visually inspected.

To avoid any contribution from the laboratory lights on the I_2_ signal, fresh films were prepared prior to each experiment under darkened conditions in the fume hood (lab lights off and a blackout curtain was used to surround the fume hood) and only red light was used during coating of films to minimize I_2_ activation; the CWFT reactor was also covered by a blackout curtain. Each type of experiment was repeated 2–4 times, and all were conducted at ambient pressure. Table 1 shows the average experimental conditions (film volume, film concentration, time) and average results (maximum ΔI_2_, % IO_3_^–^ consumed) for repeat experiments of similar lengths of time. Instances where varying the duration of the repeat experiments led to significant differences in the magnitude of results, the range of time, along with their associated range in results, is reported.

#### 2.2.1 Photochemical Experiments

**Figure 3:**
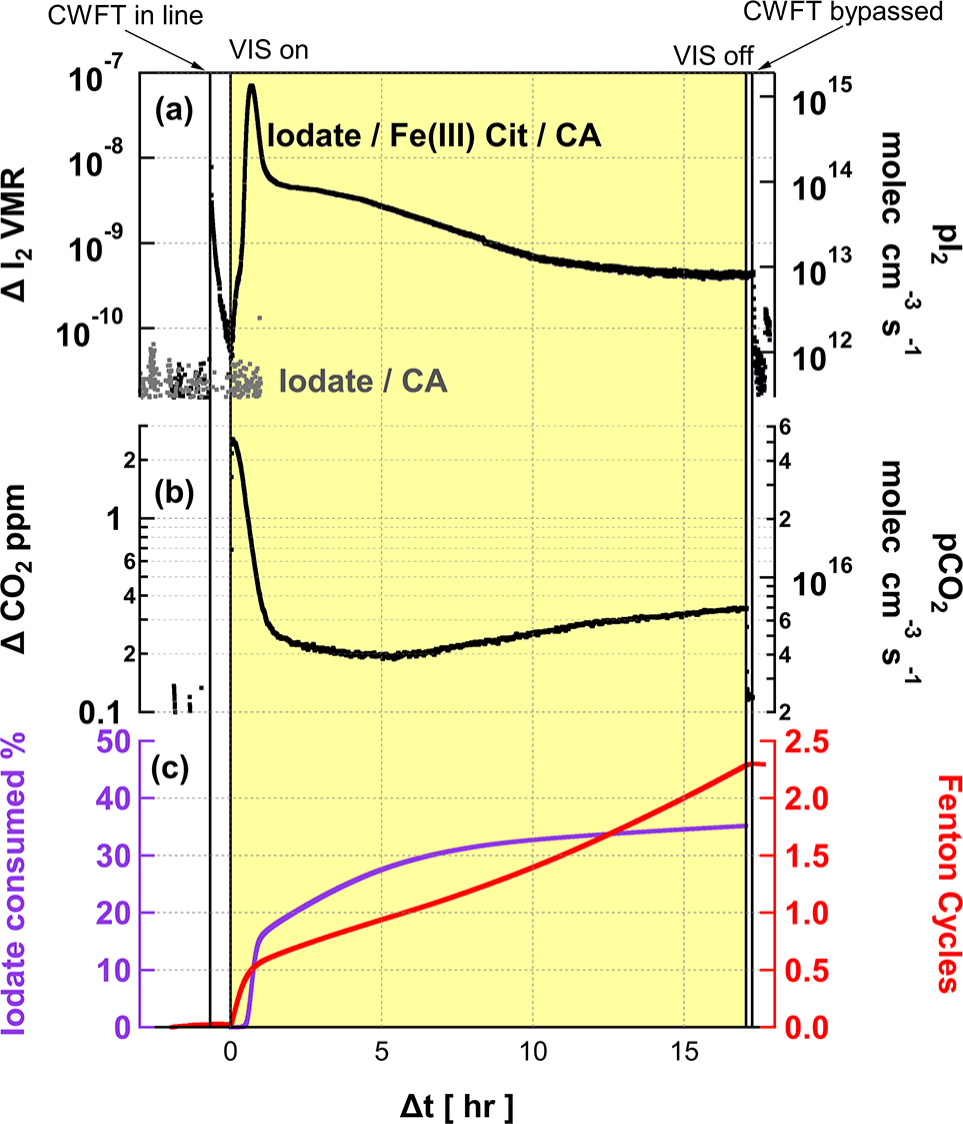
Time-series of a photochemical experiment using visible light. The black trace shows the I_2_ produced from a film containing Fe–Cit, a known photocatalyst, while the gray trace shows a case without it (a). The CO_2_ released from the photocatalyzed reaction peaks and then quickly decreases as I_2_ forms (b). The purple trace shows the percent iodate consumed to form I_2_, while the red trace shows the number of Fenton cycles tracked by CO_2_ production. Here, a Fenton cycle is defined as the ratio of integral CO_2_ to the initial amount of Fe–Cit in the film (c).

The typical measurement sequence for photochemical experiments involved the following: (1) flowing the carrier gas flow through the water bubbler; (2) placing the reactor in the “bypass mode” to insert the CWFT into the cooling jacket. In this mode, the humidified carrier gas flow is directed through the bypass line instead of through the CWFT. This allows for the characterization of our background signal, measuring any I_2_ present in the lines prior to the start of the experiment. Then, as shown in Figure 3; (3) the CWFT was placed “in line”, allowing the humidified carrier gas to flow through the CWFT. This step is marked by a steep increase in I_2_, arising from low-light activation of iodate. This was only observed when Fe–Cit was part of the matrix, potentially due to the uncontrolled reduction of Fe^3+^, even under the optimized dark conditions of the coating preparation. (4) The lamps were turned on following the decrease of I_2_ in the dark reactor. The CWFT was irradiated with visible lights and UV-A lights, separately. (5) The lamps were turned off after some time (10 min–several hours) and the reactor containing the CWFT reactor was placed in bypass mode to remove the tube.

#### 2.2.2 Dark Experiments

The typical measurement sequence for dark experiments involved the following steps: (1) flowing the carrier gas flow through the water bubbler; (2) placing the reactor in the “bypass mode” to insert the freshly prepared CWFT into the cooling jacket; (3) placing the CWFT “in line”, allowing the humidified carrier gas to flow through the CWFT and waiting for the RH level to equilibrate (10–30 min); (4) setting the CWFT in “bypass mode” to switch the carrier gas to flow through the H_2_O_2_ bubbler. As this step is usually followed by a peak in I_2_ (see Figure S1a.), we waited for the signal to come back down and remain constant for ∼10 min; (5) then, the CWFT was placed “in line”, allowing the carrier gas flow containing H_2_O_2_ to flow through the CWFT in the dark. (6) At the end of a dark experiment, the CWFT reactor was placed in “bypass mode” to remove the tube. The background signal of I_2_ present in the lines was taken as H_2_O_2_ flowed through the bypass line, i.e. while the CWFT reactor was in the “bypass mode”. The measurement used to background correct the I_2_ signal was either from prior to the start or after the end of the experiment; whichever was closest in time to when the average release of iodine was determined for an experiment, while the CWFT was “in line” (see Figure S1b.). Dark-aging + VIS experiments involved doing steps 1–5, followed by irradiation with visible light (Figure S1).

### 2.3 Instrumentation

The gas-phase products evaporating from the film were sampled by three different instruments: I_2_ was measured by the University of Colorado Light Emitting Diode Cavity-Enhanced Differential Optical Absorption Spectroscopy (CU LED-CE-DOAS) instrument;^36,37^ CO_2_ was measured by a Nondispersive IR CO_2_ monitor (NDIR, Teledyne T360U); oxygenated volatile organic compounds (OVOCs) were measured with a Vocus CI ToF using iodide as the reagent ion (I-CIMS) for a singular photochemical experiment (iodate/CA/Fe–Cit film and VIS light).

#### 2.3.1 LED-CE-DOAS

The CU LED-CE-DOAS detects I_2_ absorption at green wavelengths, its design builds on the setup described in detail elsewhere.^36,37^ Briefly, a green LED (523 nm, LED Engin LZ1–00G102) is coupled into an optical cavity consisting of two high reflectivity mirrors (*R* = 0.999960) peaking at 531 nm that are placed 86.7 cm apart, and purged with N_2_ (sample path length of 74.8 cm). The mirror reflectivity is determined by flowing He and N_2_ gas, utilizing their differences in Rayleigh scattering cross-sections as described in Thalman et al. 2014.^38^ The optical path length is wavelength dependent, and was ∼20 km near peak reflectivity. The light exiting the cavity is focused onto an optical fiber coupled to an Ocean Insight QE 65000 spectrometer (0.55 nm resolution). Spectra were collected every 15 s and analyzed using the QDOAS spectral fitting software^39^ to retrieve I_2_ slant column densities (SCDs, integrated concentration along the light path). The CE-DOAS fit settings are listed in Table S1. The correction method described in Horbanski et al., 2010,^40^ was used to account for the light path reduction due to strong I_2_ absorption at high concentrations. The method relies on absolute intensities (as opposed to the natural logarithm of the intensity ratio typical for DOAS) of the measured spectrum and reference (N_2_) spectrum as input to the least-squares fitting algorithm. With knowledge of the cavity path length, the retrieved SCDs are converted to concentrations using the Lambert–Beer’s law. The I_2_ volume mixing ratios (VMRs) are calculated by dividing the concentration by the number density of air in the cavity. A 5 min running average was calculated for the I_2_ VMRs from the 15 s spectra. The detection limit of the 5 min averaged data was ∼6 pptv for I_2_; where limit of detection (LOD) equals 3σ, with sigma being the standard deviation of I_2_ variability at constant I_2_ concentration, scattered around zero.

#### 2.3.2 I-CIMS

A Vocus 2R Long Time-of-Flight Chemical Ionization Mass Spectrometer (Vocus LToF I-CIMS, Tofwerk AG, Thun, Switzerland) from the University of Colorado was used to detect gas-phase products from a single photochemical experiment with a film composition of iodate/CA/Fe–Cit, irradiated with visible light. I-CIMS used the I^–^ reagent ion to provide real-time molecular level measurements with minimal ionization-induced fragmentation. The I-CIMS consists of two parts, the chemical ionization unit (AIM reactor^41^) and time-of-flight mass spectrometer.^42,43^ Briefly, the sample flow is continuously sampled at a constant rate of ∼2 standard liters per minute (slpm). In the ion–molecule reaction (IMR) region, the molecules of interest (M) cluster with the reagent ions (I^–^) to form negative charged adducts ([M]I^–^) via chemical reactions (M + I^–^ → [M]I^–^). Calibration of the I-CIMS was performed using I_2_ measured by the CE-DOAS to derive a calibration factor of 3.5 × 10^13^ molec. cm^–3^ ncps^–1^ for I_2_. For now, we use CIMS measurements for a qualitative detection of products other than I_2_.

#### 2.3.3 Avantes UV/Vis Light Source

The Avantes AvaLight-D(H)–S is a deuterium and halogen light source that was used to measure the absorption of a CA solution. A cuvette was filled with a 67.5 mM CA solution, spectra were collected every 0.6 ms, for 1 h. Then, the cuvette was filled with ultrapure water and used for a reference measurement. Figure 2c. shows how the absorption cross-section of CA increases in the UV wavelength range, from the start of the measurement, after 30 min of sampling, until the end of the hour-long sampling period.

### 2.4 Chemicals

The following chemicals were used to create the iodate films: Sodium iodate (laboratory reagent grade, Fischer Chemical), ABS (≥98.5%, Carl Roth GmbH + Co. KG), BTCA (99%, Sigma-Aldrich), CA (≥99.5%, Sigma-Aldrich), Fe–Cit (technical grade, Sigma-Aldrich), ATD (ultrafine, Powder Technology Incorporated), and Fe_2_O_3_ (99.945%, Alfa Aesar). The following were used for cleaning of the glass tubes: Ethanol (VWR Chemicals BDH), acetone (Sigma-Aldrich), HCl (Acros Organics) and NaOH (Sigma-Aldrich).

### 2.5 Calculations

#### 2.5.1 I_2_ and CO_2_ Production Rates

Calculated ΔI_2_ and ΔCO_2_ are background corrected signals. A constant background I_2_ signal is determined by taking the average when the CWFT is bypassed (see details in Sections 2.2.1 and 2.2.2). A constant background of CO_2_ is determined by taking the average signal when the CWFT is bypassed before and after the experiment.
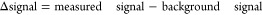
1

Calculation of production rates pX (molecules cm^–3^ s^–1^), X = I_2_ or CO_2_
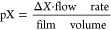
2

Where Δ*X* is the background corrected concentration of the species in molecules cm^–3^, the flow rate is in units of cm^3^ s^–1^, and film volume in cm^3^.

Calculation of integral *X* molecules, *X* = I_2_ or CO_2_, was done by calculating the number of molecules in the time interval (in seconds) that each data point *i*, was collected, then, summing these for the duration of the experiment.
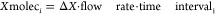
3
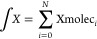
4

The percent iodate consumed % (to produce I_2_), %IO_3-consumed_ was calculated as follows
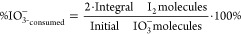
5

The photolysis of Fe–Cit under UV–vis light leads to the formation of Fe^2+^ and the decarboxylation of the central carboxyl group in Fe–Cit, releasing CO_2_.^24,26,46^ A Fenton cycle refers to the oxidation of Fe^2+^ to Fe^3+^ by reactive oxygen species, ROS (i.e., O_2_, HO_2_, H_2_O_2_, RO_2_), followed by the reformation of Fe–Cit in the presence of CA. The newly formed Fe–Cit molecule can photodissociate and go through the cycle again. Measurements of the Fe(II) quantum yield of Fe–Cit photolysis provide a range of values reaching down as low as 0.2, depending on solution pH, concentration, Fe to citrate ratio, and available oxidants.^26,47^ For simplicity, we assume that the quantum yield for the photodissociation of Fe–Cit is close to unity in UV-A and VIS light, and that for every Fe–Cit molecule that dissociates, a CO_2_ molecule is produced. Here, we define an equivalent number of Fenton cycles that occurs within a system as follows
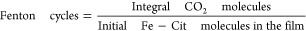
6

Given the uncertainties in Fe(II) and CO_2_ yields, we consider this a semiquantitative proxy for the photochemical cycling of iron.

#### 2.5.2 Film Conditions

The volume of the film in the CWFT is dependent on its water content and it is set by the water activity of the film. This is equal to the relative humidity of the carrier gas flow when thermodynamic equilibrium is reached. The hygroscopic properties of the film components are considered for approximation of the water content of the film. The mass of water is then used to calculate the mass of the film, and this is divided by the estimated density of the film to give the film volume. Detailed calculations for the determination of film volumes are found in the Supporting Information.

Briefly, the volumes for iodate/CA and iodate/CA/Fe–Cit films were calculated by considering the following assumptions about the density of each film: (1) the density of the film is calculated as a weighted mean of the density of the solutes, where the density of CA was determined as in Lienhard et al., 2012;^28^ (2) the density of Fe–Cit is directly proportional to the density of CA; and (3) the contribution of NaIO_3_ is negligible (see Supporting Information text for details). The well-characterized hygroscopic properties of CA and the measured water activity of 1 M iron(III) citrate solution by Dou et al., 2021^24^ were used to calculate the mass fraction of water.

AIOMFAC-web (http://www.aiomfac.caltech.edu), a thermodynamic activity coefficient model, was used to determine the water content for the iodate/ABS and iodate/BTCA films.

## 3 Results and Discussion

The photoactive Fe–Cit molecule consists of a central iron atom and citrate ligand, which is the triply dissociated form of CA, a hydroxyl-tricarboxylic acid. The photochemical reaction scheme of Fe–Cit is characterized by UV–vis light (λ < 500 nm) inducing ligand-to-metal charge transfer (LMCT) yielding Fe^2+^ and citrate radical,^26^ followed by decarboxylation of the citrate radical at the central carbon near the OH group, releasing carbon dioxide, CO_2_, (as shown in Figure 3b), and the ketyl radical, which in the presence of O_2_ produces ROS that support Fenton reactions.^24,48,49^ The OH radicals produced from Fenton reactions can react with CA present (R4). Additionally, in the presence of CA, the oxidation of Fe^2+^ to Fe^3+^ leads to the reformation of Fe–Cit (R5).^24,26^ This reaction scheme is summarized by reactions R1–R5.
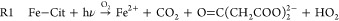

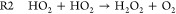

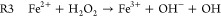

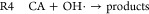

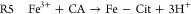


As seen in Figure 2c., CA can generate a chromophore under UV-A light, possibly due to the oxidation of the hydroxyl group to a carbonyl. To prevent such uncontrolled formation of a chromophore, we chose BTCA as a second organic matrix, as it is missing this hydroxyl group-oxidation site. ABS was the inorganic matrix of choice due to the prevalence of sulfate in inorganic aerosol. The bisulfate salt was chosen to have similar pH in the film as with the organic acids.

Our results demonstrated that visible light triggers iodate reduction (release of I_2_) from inorganic and organic fresh films with the addition of a photocatalyst (i.e., Fe–Cit or dust proxy), and it does so more efficiently than UV-A light (Sections 3.2). It was found that the dark reaction of H_2_O_2_ and iodate can activate over 30% of iodate into I_2_, given extended reaction times (Section 3.3). Interestingly, aging of iodate films in absence of iron in the dark with H_2_O_2_, prior to irradiation with VIS light, enhanced the release of I_2_ under subsequent VIS irradiation of the aged films to levels attributed to photocatalyzed reactions in fresh films containing iron. Furthermore, this indirect effect of H_2_O_2_ suggests the production of a chromophore, and was observed to have a greater impact on iodine activation than the direct effect of H_2_O_2_, i.e. dark reduction of iodate (Section 3.4).

The photolysis lifetime of molecular iodine, I_2_, in the gas phase was calculated using the actinic flux of the 3 UV-A and 4 VIS lamps in the photoreactor as 9.5 min and 45 s, respectively. With a residence time of 1.2–1.4 s in the CWFT, we do not expect I_2_ evaporated from the film to be photolyzed by the lamps. Dissolved I_2_ that remains in the film, however, is subject to photolysis by the lamps, given that the photolysis lifetime of I_2_ in the aqueous phase is 1.6 min and 29 s in UV-A and VIS light, respectively. This means that the I_2_ evaporated from the film, which is sampled by the CE-DOAS, is a conservative estimate of the I_2_ produced, and highlights the importance of studying thin films to allow for product evaporation (over bulk solutions). We observe an enhanced release of I_2_ from irradiated aged films (exposed to H_2_O_2_ in the dark prior to irradiation), compared to irradiated fresh films and to films exposed to H_2_O_2_ in the dark. This suggests that the enhancement of I_2_ observed in irradiated aged films is related to the formation of chromophores in the dark.

### 3.1 Comparison of VIS and UV-A Photochemical Experiments

**Figure 4:**
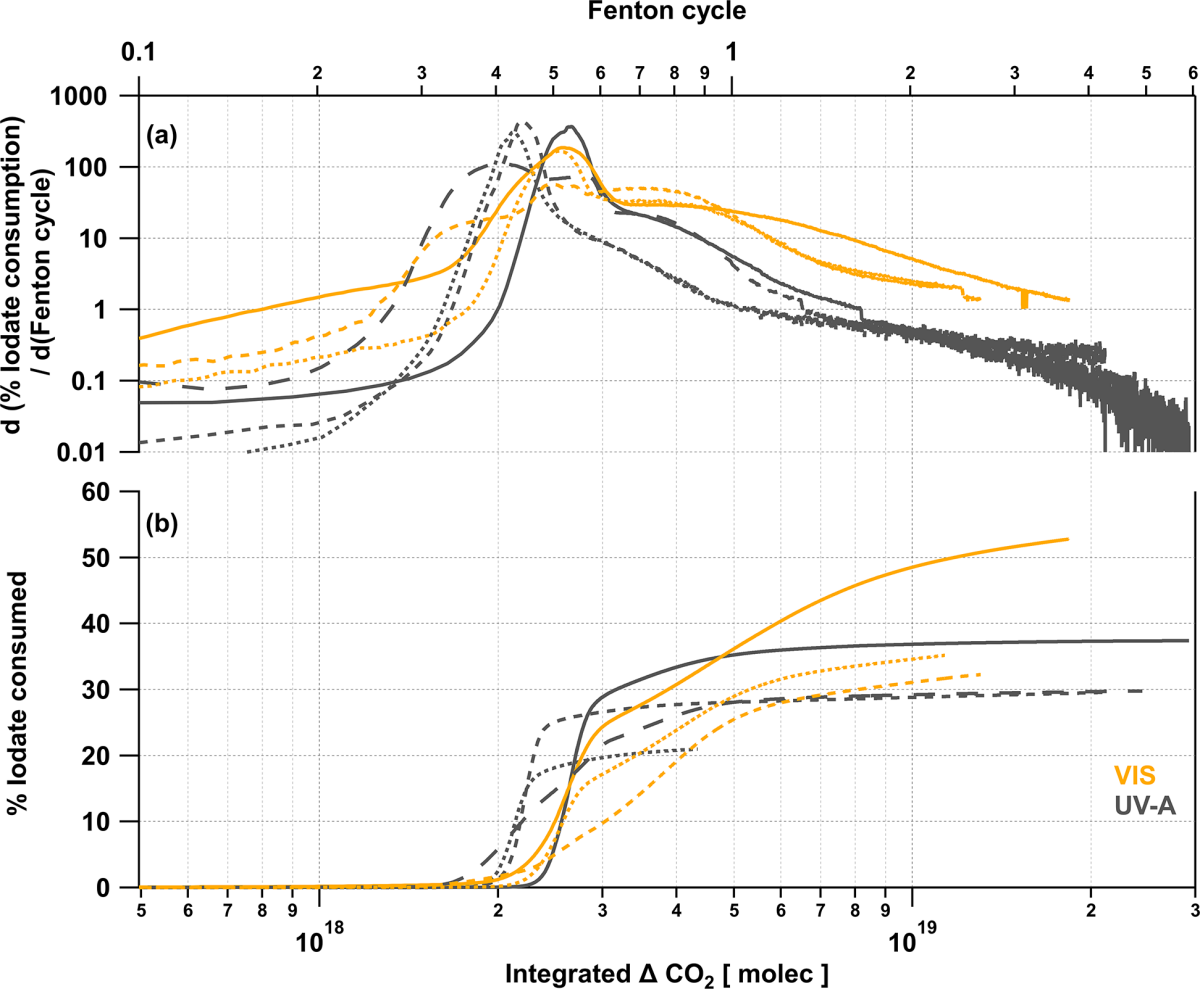
Comparison of the visible light (yellow) and UV-A light (gray) – photocatalyzed reduction of iodate. The rate of change at which iodate is consumed to form I_2_, with respect to Fenton cycles, is greater under visible light than UV-A light (a). Visible light results in a larger percentage of iodate consumed to form I_2_, as a function of integrated CO_2_ molecules (or Fenton cycles) (b).

To compare the photochemical processing of iodate/CA/Fe–Cit under UV-A and VIS light, we first consider the maximum ΔI_2_ released (Table 1) under both types of light. While UV-A leads to a higher instantaneous maximum ΔI_2_ release (up to 235 ppbv I_2_), the percent iodate consumed over the whole period is higher for the VIS experiments. The visible irradiation of fresh films containing Fe–Cit results in 30–50% consumption of iodate in the film to form I_2_, compared to the 21–32% under UV-A light. A closer look at the percent iodate (to produce I_2_) consumed per Fenton cycle provides a measure of the reaction’s efficiency over time. Figure 4a. shows the rate of change of percent iodate consumed per Fenton cycle for repeated VIS and UV-A experiments. The rate of change is higher for the VIS experiments at the beginning of the reaction and also toward the end of the experiment. Figure 4b. shows that VIS light is also sustaining a larger percent iodate consumed (i.e., integral release of I_2_) after a single Fenton cycle. The calculated photolysis rate of Fe–Cit under visible lamps was 0.0011 s^–1^, a factor of 7 lower than with UV-A lamps (0.0074 s^–1^). Despite the larger photolysis rate of Fe–Cit in UV-A light, a higher percentage of iodate is reduced to I_2_ under VIS light.

In effect, VIS light activates iodate more efficiently than UV-A light for fresh films containing the photoactive compound, Fe–Cit. This, of course, is solely based on the conversion of iodate to I_2_. The chemical speciation of products was observed for this system under VIS light (see Section 3.5), and I_2_ was found to be the major product. Presumably, this is also the case for UV-A light, however, more experiments are needed to confirm this assumption, as the speciation of iodinated products, e.g. iodinated organic volatile organic compounds, I-OVOC, under UV-A light could differ from what was observed with visible light.

**Figure 5:**
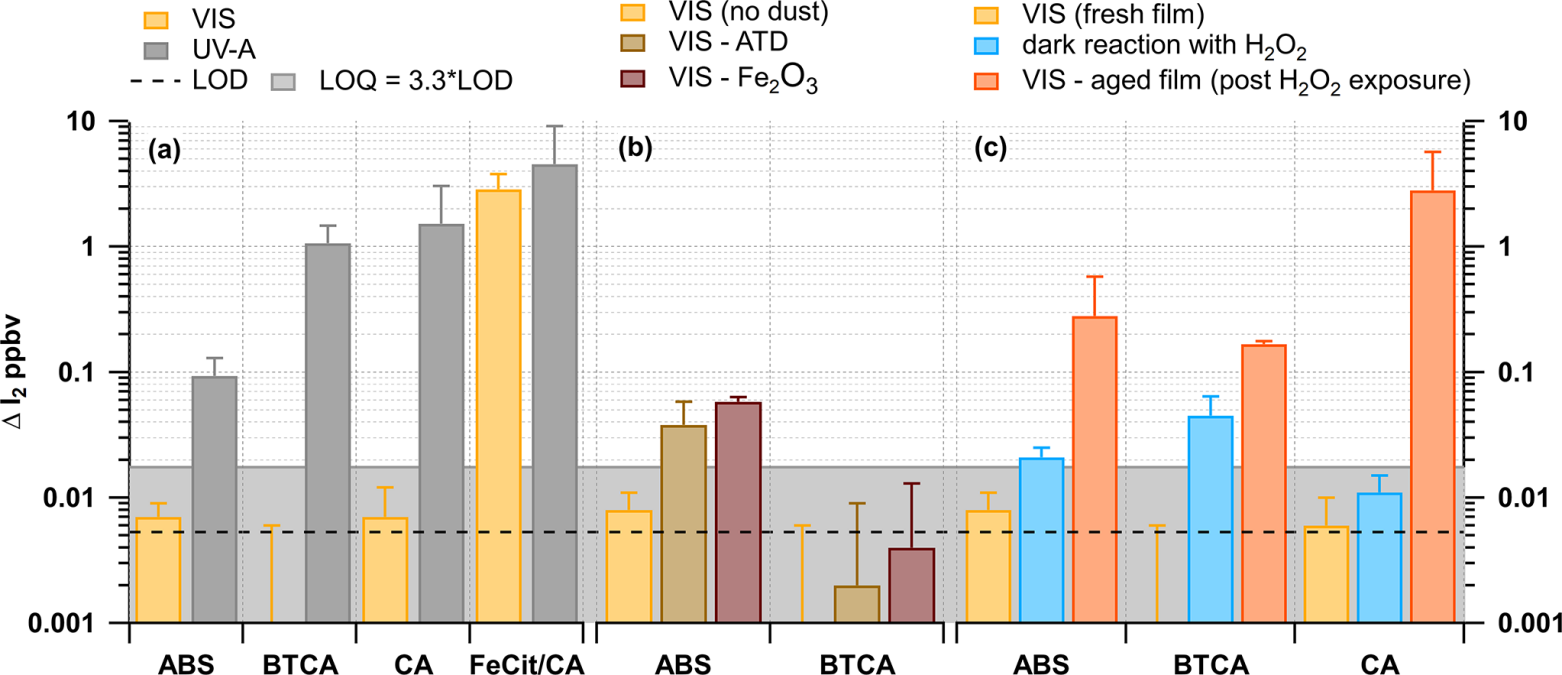
ΔI_2_ (ppbv) released from iodate is calculated by subtracting the background I_2_ from the average signal. Adding a known photoactive compound, Fe–Cit, results in the largest release of I_2_ under visible light (a). Dust proxies in aqueous iodate films enhance the I_2_ released from visible light irradiation (b). I_2_ produced from irradiated films aged with H_2_O_2_ (orange) is greatly enhanced compared to dark (blue) and photochemical (yellow) experiments using fresh films (c). The length of the error bars is a 95% confidence interval for the mean.

Figure 5 summarizes the average ΔI_2_ released for each type of experiment, values above the LOD are considered to be significant, but only values above the limit of quantification (LOQ = 3.3 × LOD), represented by the gray shaded region, can be quantified with high confidence. To compare the amount of I_2_ released from the irradiation of each type of iodate film matrix under VIS and UV-A light, the average ΔI_2_ (ppbv) released during the irradiation period, for repeat photochemical experiments, are shown in Figure 5a. It should be noted that the lengths of irradiation time between the different matrices differ (see Table 1). Nevertheless, the big picture remains; a significant amount of I_2_ is released from the iodate/CA/Fe–Cit film under both VIS and UV-A light, this is expected due to the presence of a photoactive compound. For cases where a chromophore was absent (iodate/ABS, iodate/BTCA, and iodate/CA), there was little to no detectable amount of I_2_ released under VIS light, the small I_2_ signals observed may be due to wall effects. Figure 5b. shows that the BTCA system under visible light has the lowest backgrounds of all systems. We chose BTCA to establish the absence of analytical artifacts, and to link the formation of I_2_ to the chemical differences in the chosen matrices, rather than artifacts of transition metal chemistry due to impurities on glassware. Under UV-A light, however, these type of analytical artifacts were not possible to avoid, and a substantial release of I_2_ was observed in absence of an added chromophore, both in the inorganic and organic films. We again refer to Figure 2c., indicating building up of additional absorbance during repeated UV–vis measurement of CA solution over extended times. This could be due to carbonyl formation in CA, and organic contaminants in ABS^50^ producing chromophores such as aldehydes or imines. However, the exact reasons why this is observed are currently unknown.

### 3.2 Photochemical Experiments: Dust Proxies

To assess the effect of dust proxies in the film, we compare the average ΔI_2_ (ppbv) released for the cases where a chromophore was absent, and the I_2_ released with added dust proxies (i.e., ATD or Fe_2_O_3_). ATD is a mixture of metal oxides, including titanium dioxide, TiO_2_, and hematite, Fe_2_O_3_, both of which are known photochemical sources of radicals.^51^ We expected some enhancement in the amount of I_2_ released from matrices containing dust proxies due to their photoactive properties. TiO_2_ absorbs UV photons, while Fe_2_O_3_ absorbs in the UV–vis range (up to 600 nm),^52^ generating electron–hole pairs that lead to formation of radicals, e.g. OH, HO_2_.^53^ The photocatalytic properties of Fe_2_O_3_ are characterized by the formation of superoxide radicals (O_2_·^–^) and hydroxyl radicals (OH·), with its performance limited by the recombination of electrons and holes at the surface.^52^ We also note that in both ABS and BTCA, some dissolution of Fe from the different iron bearing minerals contained in ATD is possible, leading to Fe(III) hydroxy or carboxylate complexes, which could then act as photocatalysts, as in the experiments with Fe–Cit. Figure 5b. shows how the addition of a dust proxy (ATD or Fe_2_O_3_) leads to a detectable enhancement of I_2_ released in the inorganic matrix (ABS), and adding Fe_2_O_3_ resulted in a larger increase than adding ATD. Dust proxies added to the organic matrix (BTCA) resulted possibly in a marginal increase in I_2_ released, albeit, at levels close to or below the detection limit of the CE-DOAS. From Table 1, we see that adding dust proxies to the film leads to an increase in the percent of iodate reduced from the inorganic matrix (ABS) while no effect was observed for the organic matrix (BTCA), possibly due to efficient scavenging of radicals by BTCA. These findings provide laboratory evidence in support of dust activation of iodate as iodine and are compatible with recent field observations that have detected elevated IO radicals inside lofted dust layers in the free-troposphere.^14,54^

### 3.3 Dark Experiments: Iodate Reduction by H_2_O_2_

The molar ratio between H_2_O_2_ and iodate was varied for the iodate/CA matrix, these results are summarized in Table S3. It was found that increasing the molar ratio between H_2_O_2_ and iodate from 2.1:1 to 2.8:1 led to an increase in the percent iodate consumed by 1 order of magnitude (from 0.035–0.43%) over the course of 16 h of dark exposure to H_2_O_2_. Increasing the molar ratio further (from 2.8/1 to 14/1) led to a smaller increase, from 0.43–2.45%, after 16 h of dark exposure to H_2_O_2_. From the 67 h dark experiment in Table 1, we observed that the percent iodate consumed from the dark reaction of iodate and H_2_O_2_ can reach levels comparable to the photocatalyzed reduction of iodate, if given enough time (∼37%).

For each of the matrices (ABS, BTCA, and CA), it was found that the dark reaction of H_2_O_2_ and iodate in the film released a larger amount I_2_ than from the photochemical activation with VIS light. Figure 5c. shows that the average amount of I_2_ released from the dark reaction of H_2_O_2_ and iodate films in a matrix of either ABS, BTCA, or CA (blue bars) was enhanced over background by a factor of 2–6 (the background experiment here is the VIS irradiation of a fresh film). The I_2_ observed in the dark varied between the matrices; the iodate/BTCA film showed a significant and quantifiable amount of I_2_ produced (∼40 pptv), iodate/ABS formed a lesser amount (∼20 pptv), and the iodate/CA film produced I_2_ levels below quantification. Over extended reaction times, the dark reaction of iodate with H_2_O_2_ at high concentrations, can activate significant I_2_, as is reflected in ∼3 orders of magnitude higher %IO_3-consumed_ compared to the background experiment (Table 1).

The dark reaction of iodate with H_2_O_2_ is an important initiation step for iodate reduction and iodine oscillation reactions.^16,17^ As described in Schmitz and Furrow 2016,^55^ the reduction of iodate by H_2_O_2_ in acidic solution is a complex multistep process. Iodate is first reduced to iodous acid, HIO_2_, followed by reduction to hypoiodous acid, HOI. HOI reacts with H_2_O_2_ to produce HOOI, and this peroxide decomposes to yield iodide, I^–^. HOI then reacts with I^–^, resulting in the formation of I_2_. The reduction of iodate by H_2_O_2_ has been well studied in acidic bulk solutions; with proton concentrations ranging from 0.01 to ∼0.2 M, and under low (<0.1 M) and high (>0.1 M) concentration of H_2_O_2_, at 0.099 M iodate.^16,17^ Assuming that I_2_ formation is limited by the kinetics of this reaction, our results support the results from Bray and Liebhafsky,^16^ that find the reduction of iodate by H_2_O_2_ is more easily accomplished at lower [H^+^]; as the highest production rates of I_2_ were observed for BTCA, the matrix with the lowest [H^+^] (Table S2). In this work, we test if the known kinetics of this reaction in bulk solutions is applicable also in 2 μm thick aqueous films at high solute strength that serve as aerosol proxies at atmospherically relevant iodate concentration. The factor difference between the theoretical and observed production rate of I_2_ (based on the Bray–Liebhafsky kinetics approximation in Schmitz and Furrow 2012,^17^ under low H_2_O_2_ concentration, [H_2_O_2_] < 0.1 M, see Supporting Information for calculation details), for each matrix is found in Table S2. This factor ranges between 2 to 6 for the iodate/CA and iodate/ABS matrices, with a molar ratio of ∼2.8:1 (H_2_O_2_: iodate). The activation of iodine by H_2_O_2_ is similar or lower than that expected in dilute solutions. For the iodate/BTCA matrix, however, the observed production rate of I_2_ is only slightly larger, and compares more closely to the theoretical production rate (by a factor of 1.8–2.9). The reacto-diffusive length of H_2_O_2_ is larger than the film thickness by 5 orders of magnitude (see Table S2), i.e. we can expect H_2_O_2_ to be homogeneously distributed throughout the film and not significantly depleted due to reaction with IO_3_^–^ in the films.^56^ Thus, H_2_O_2_ cannot explain these differences and other factors must be at play.

In the simple kinetic analysis above, we have assumed ideal solution conditions. The high solute strength may lead to substantial alterations: activities may significantly differ from concentrations and may also affect the solubility, meanwhile, ionic strength may significantly affect the kinetics. Salts decrease the solubility of most species due to “salting out”. With respect to H_2_O_2_, salting effects are species-dependent; past studies have found the presence of ammonium sulfate leads to increasing the solubility of H_2_O_2_^57^ by up to a factor of 2 compared to pure water,^58^ while hydrogen ions from acids decrease the solubility of H_2_O_2_.^57,59^ To estimate an approximate magnitude of the salting effect with respect to H_2_O_2_, we assume a salting constant of 0.25 m^–1^ and salt concentrations from Table 1. Under this assumption, salting-out of H_2_O_2_ can explain a factor ∼5.6 reduction in the Henry’s Law partitioning coefficient assumed in the theoretical calculation. There is a need for further laboratory experiments to test this hypothesis.

### 3.4 Dark-Aging + VIS Experiments: Production of a Chromophore

Figure 2c. displays the absorption of a CA solution at UV-A and VIS wavelengths. An increase in the absorption of light by CA with sampling time is observed more prominently in the UV-A region than in the VIS. Figure 5a. clearly shows this distinction and its effect on I_2_ evaporated from irradiated films; with significant I_2_ being released in the absence of a photoactive compound, only under UV-A light. This is presumably due to impurities in the system (e.g., glassware); along with previously mentioned uncontrolled production of chromophores at UV-A wavelengths; e.g. oxidation of CA, followed by photolysis of the resulting carbonyl product, leading to further reactions. Films aged with H_2_O_2_ in the dark were followed by VIS light irradiation, eliminating any unconstrained effects tied to impurities and UV-A light. Figure 5c. demonstrates that the I_2_ released from irradiated aged films was greater than that from irradiated fresh films (in absence of a photoactive compound), or fresh films exposed to H_2_O_2_ in the dark. Table 1 shows that over half of the iodate can be consumed to produce I_2_ with prolonged irradiation of aged films. The heightened release of I_2_ from irradiated aged films (with VIS light) suggests the production of a chromophore-containing compound. In Figure 5c., the enhancement of I_2_ evaporated from the films is seen in both aged inorganic (ABS) and organic matrices (BTCA and CA); the enhancement ranges between a factor of 4 (iodate/BTCA film), and 2 orders of magnitude (iodate/CA film). The choice of BTCA as a second organic matrix attempts to disentangle the role of uncontrolled chromophores resulting from potential photochemical oxidation of CA (see Figure 2c.) on the results observed. We can assume some of the increase in I_2_ during the irradiation of the aged iodate/CA film to be tied to the aforementioned photoactivity of CA. Interestingly, the enhancement observed for the inorganic film (iodate/ABS) is about an order of magnitude difference, lying in the middle of the enhancement range. Thus, we can surmise that the dark reaction of iodate and H_2_O_2_ leads to the production of inorganic and/or organic chromophore products, leading to enhanced levels of I_2_ upon irradiation with visible light, between a factor of ∼4 and an order of magnitude greater than I_2_ released from the dark reaction of iodate and H_2_O_2_. The chromophore(s) produced could be a combination of organic and inorganic species, also including the multitude of iodine species produced as intermediates to I_2_ formation. More experiments are needed to identify the chemical composition of the chromophore(s) and determine if these are iodinated compounds.

The direct and secondary effects of H_2_O_2_ (due to chromophore production) were assessed by comparing the percent iodate consumed from the dark reaction of H_2_O_2_ and fresh iodate/CA films to that from the irradiated aged films. Table S3 shows how increasing the length of time that H_2_O_2_ reacts with iodate in the dark by a factor of ∼30 only leads to a factor of ∼3 increase in %IO_3-consumed_. Meanwhile, increasing the irradiation time of aged films, by a factor of ∼40, results in a factor of ∼30 increase in %IO_3-consumed_. Thus, the indirect effects of H_2_O_2_, i.e. chromophore production, appears to dominate over the direct effect of H_2_O_2_ on iodine activation.

### 3.5 Photochemical Experiments (VIS): Chemical Speciation

**Figure 6:**
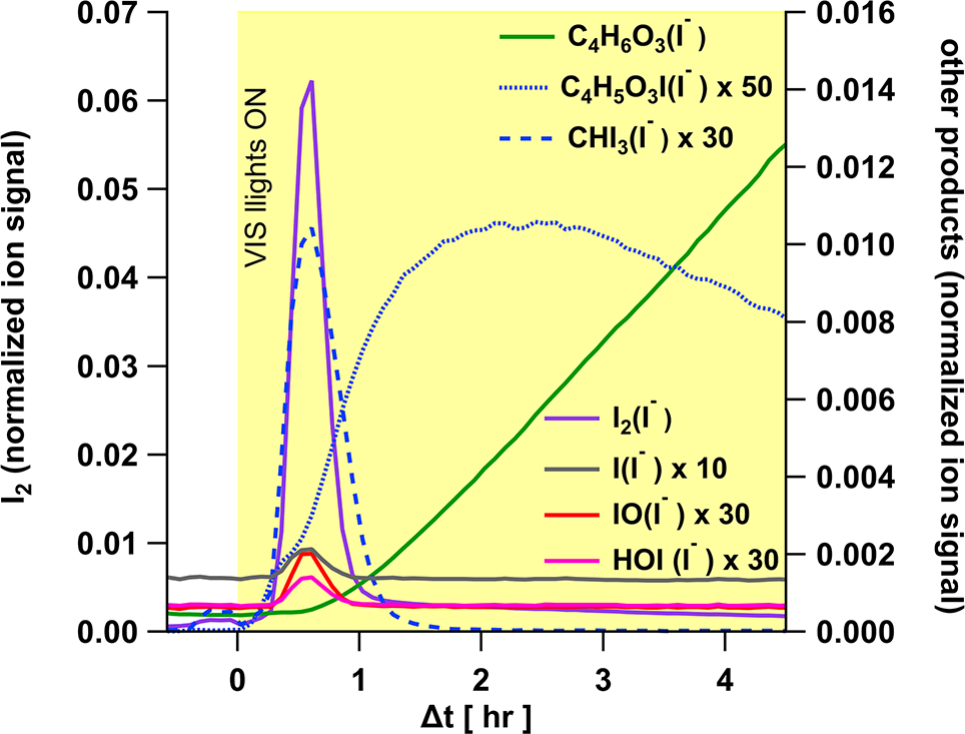
Time-series of volatile products released from the photocatalyzed reduction of an iodate/CA/Fe–Cit film irradiated with visible light. The three types of products shown are (a) inorganic iodine, mostly as I_2_ (purple), (b) OVOC fragments of the citrate ligand (green) initiated by Fe–Cit photolysis, and (c) I-OVOC (blue).

Figure 6 shows a time-series of products from the photocatalyzed reduction of iodate under VIS light, as detected by I-CIMS. The iodate/CA/Fe–Cit film released three kinds of products: (a) inorganic iodine species, (b) oxygenated volatile organic compounds (OVOC), and (c) iodo-OVOC (I-OVOC). The major inorganic species released from the film was molecular iodine (shown in purple) I_2_·I^–^ (*m*/*z* 380.714), with HOI·I^–^ (*m*/*z* 270.812), I·I^–^ (*m*/*z* 253.809) and IO·I^–^ (*m*/*z* 269.804) observed as minor signals.

The OVOC C_4_H_6_O_3_·I^–^ (*m*/*z* 228.936) was a major organic product (shown in green), it increased steadily after the VIS lights were turned on, likely a degradation product of the citrate ligand initiated by Fe–Cit photolysis. The photolysis of Fe–Cit complexes are expected to yield 3-ketoglutarate (C_5_H_4_O_5_)^60−62^ or 3-ketoglutaric acid (or acetone dicarboxylic acid, O=C(CH_2_COOH)_2_) from the following steps: (1) citrate radical production, (2) decarboxylation of the citrate radical at the central carboxyl group, producing the ketyl radical, and (3) oxidation of the ketyl radical’s alcohol group by reaction with O_2_ via H-transfer.^24^ It has been shown that 3-ketoglutarate can be converted to acetoacetate (C_4_H_5_O_3_) by nonredox decarboxylation rearrangement, and this reaction can be catalyzed by divalent metal ions,^62^ like Fe^2+^. It is possible that the C_4_H_6_O_3_·I- (*m*/*z* 228.936) detected is similarly formed from the decarboxylation of 3-ketoglutaric acid, facilitated by Fe^2+^, yielding acetoacetic acid. The iodo-OVOC detected (shown in blue) include: C_3_H_4_OI_2_·I^–^ (*m*/*z* 436.740, moderate), C_3_H_3_OI_3_·I^–^ (*m*/*z* 562.636, moderate), C_4_H_5_O_3_I·I^–^ (*m*/*z* 354.833, minor), possibly an iodinated form of acetoacetate, and CHI_3_·I^–^ (*m*/*z* 520.626 minor).

Soluble organic iodine (SOI) is a major fraction of particulate iodine in the marine boundary layer.^63^ Organic iodine has been attributed to biogenic sources (marine algae) and abiotic sources via reactions between methyl radicals and I atoms,^64^ and between I_2_/HOI and dissolved organic matter.^65^ Detection of inorganic and organic iodine from the iodate/CA/Fe–Cit film with VIS light demonstrates that the irradiation of aerosols containing iodate and iron–carboxylate complexes are another source of abiotic volatile organic and inorganic iodine. Moreover, the production of I–OVOCs observed as minor products from the reaction support field observations of SOI making up the majority of iodine in aged particles, while inorganic iodine species accounts for the majority composition of newly formed particles.^66^

### 3.6 Experiment Variability

#### 3.6.1 Photochemical Experiments

Iodate/ABS, Iodate/BTCA, and Iodate/CA films exposed to VIS light resulted in minimal and constant I_2_ production. The temporal production of I_2_ from iodate/ABS and iodate/BTCA films under UV-A light was different between repeat experiments; some showed a gradual increase in I_2_ (∼30 min -1 h until reaching a maximum I_2_ signal), while others showed a quick response to turning on the lights (peak I_2_ within ∼5 min). Iodate/CA films exposed to UV-A light showed a gradually increasing signal, with I_2_ peaking several hours (3–7 h) after UV-A lights were turned on. The I_2_ production from iodate/CA/Fe–Cit films irradiated with VIS or UV-A was similar in overall shape. They had a characteristic peak of I_2_ following turning on lights. As seen in Figure 3, the maximum was followed by a shoulder, the prominence of which varied between the different repeat experiments.

#### 3.6.2 Dark Experiments: Iodate Reduction by H_2_O_2_

The reaction of iodate with H_2_O_2_ is a complex system. The exact mechanism by which the reaction proceeds was not determined for the experiments conducted. The observed temporal profile for I_2_ production varied with different experimental conditions (e.g., concentration of H_2_O_2_). Under low H_2_O_2_ concentration experiments, [H_2_O_2_] < 0.1 M, the production of I_2_ resembles a step function, with a generally constant signal of I_2_ being produced throughout the experiment (disregarding the initial increase of I_2_ observed at the beginning of the experiment, due to line conditioning effects, i.e. residual I_2_ in the lines becomes mobilized with humidified carrier gas). Under high H_2_O_2_ concentration experiments, [H_2_O_2_] > 0.1 M, however, there is accelerated I_2_ production, its signal increasing with time. This difference in the temporal evolution of I_2_ suggests that there are different mechanisms of iodate reduction at play, depending on the concentration of H_2_O_2_ employed. Further laboratory experiments are needed to determine whether iodate reduction by H_2_O_2_ is the rate limiting step for I_2_ production.

### 3.7 Atmospheric Implications

Studies have found that iodate easily undergoes photolysis at shorter wavelengths in the UV region, λ < 300 nm, and have found that the photodissociation of iodate is not expected at visible wavelengths.^13,22^ In the troposphere, light at the shorter wavelengths (λ < 320 nm) is mostly absorbed by stratospheric ozone, while UV-A and VIS wavelengths are able to reach the Earth’s surface. Here, we find a chemical pathway in which visible light is sufficient for photochemical reduction of iodate, efficiently releasing I_2_ in the presence of Fe–Cit, an Fe(III)-carboxylate complex. With knowledge of the chemical speciation of products from this type of photocatalyzed reaction, we can expect most of the iodine activated to be inorganic (I_2_) with minor products being other inorganic species (IO, I, and HOI), OVOCs and I–OVOCs.

Atlantic marine aerosol samples show iodate as a major component in coarse aerosols containing unpolluted sea spray or mineral dust, likely arising from the uptake of HIO_3_ onto alkaline dust surfaces.^67^ With field observations connecting iodine species with mineral dust,^14,67^ the abundance of iron, and the photoactive nature of iron-containing species such as iron–carboxylate complexes and Fe_2_O_3_, photocatalyzed reduction via visible irradiation of organic iodate aerosols with photoactive iron, is a relevant pathway to consider adding to chemical models.

Also relevant to consider in the chemical cycling of iodine is the enhanced release of I_2_ from irradiated films aged with H_2_O_2_ in the dark (i.e., chromophore production). An important source of H_2_O_2_ in the atmosphere is the recombination of HO_2_ radicals.^68^ H_2_O_2_ can enter the aerosol phase from both gas-phase transfer or direct photochemical production in the condensed phase (i.e., photochemistry of Fe(III)-complexes). H_2_O_2_ production in both the daytime and night-time posits iodate containing aerosols reacting with H_2_O_2_, followed by efficient iodine activation by visible light during the day.

## 4 Conclusions

A series of coated-wall flow tube (CWFT) experiments revealed that visible light is sufficient for activating aqueous iodate to the gas phase, reducing it to molecular iodine, I_2_. This is most significant when a photocatalyst is added to the film, but also observed for chromophores produced within films. We found that dust proxies enhance the release of I_2_ under visible light, providing laboratory evidence in support of field measurements of elevated IO radicals in elevated dust layers in the free troposphere.^14^ The chemical speciation measurements confirm for a single photochemical experiment - a film containing a photoactive compound, iron(III) citrate and using visible light - that molecular I_2_ is a major product evaporating from the films. I_2_ formation requires the reduction of two IO_3_^–^ molecules; and in effect this multiphase reduction pathway destroys 6 ozone molecules per I_2_ molecule formed. Qualitative results also show the formation of oxygenated volatile organic compounds (OVOCs) and iodinated OVOCs among other products formed that warrant further investigation. Interestingly, the photocatalyzed reduction of iodate under visible light results in a larger integral release of I_2_ per Fenton cycle; i.e., running the system under softer conditions leads to a more efficient activation of iodine. Furthermore, exposure of the fresh films to H_2_O_2_ in the dark enhances the release of I_2_ following visible irradiation of these aged films, which is not observed for fresh films. Our results suggest that the dark reaction of iodate and H_2_O_2_ results in the formation of a chromophore-containing product that enhances iodate activation under visible light compared to conditions when no photocatalyst is present. The CWFT experimental results demonstrate the difficulty of studying the atmospheric iodine recycling system. While significant uncertainties remain, particularly regarding the kinetics and mechanisms by which iodate is reduced, the following conclusions can be drawn: the recycling of iodate occurs under different conditions, with I_2_ being a major product from the photocatalyzed reduction of iodate with visible light. For the dark reduction of iodate by H_2_O_2_, the concentration of H_2_O_2_ impacts the kinetics of I_2_ production. The results from this work suggests that the reduction of iodate by H_2_O_2_ be included for modeling iodine recycling from atmospheric aerosols to the gas phase as I_2_. More laboratory studies with time-resolved composition analysis of products are needed to answer emerging queries; like potential salting effects of H_2_O_2_ in different aerosol proxy matrices, and the chemical speciation of products for VIS and UV-A irradiation of fresh vs aged films. Still, much has been learned about the transformations of atmospheric iodine; the formation mechanism of iodic acid (HIO_3_),^9^ and knowledge of rapid new particle formation by iodine oxoacids^6,7^ now links iodine sources to particulate iodate. The efficient recycling of iodate released as I_2_ and organic iodine species from particle proxies closes a catalytic reaction cycle under which iodine oxoacids can form particles. Atmospheric models should be expanded to account for the release of I_2_ from particulate iodate by visible light, a process induced by photoactive compounds; in dust, in the form of iron carboxylates, or arising from aged aerosols reacted with H_2_O_2_.

## Supporting Information Available

Additional details of film volume calculations and comparison of theoretical and observed kinetics (PDF)
