# Supplementary material for: Iodine Activation from Iodate Reduction in Aqueous Films via Photocatalyzed and Dark Reactions
Source: ACS Earth Space Chem. 2024 Dec 3;8(12):2495–508. doi: 10.1021/acsearthspacechem.4c00224 (PMC11664648; doi:10.1021/acsearthspacechem.4c00224)
Supplement: Supplementary file 1 — sp4c00224_si_001.pdf [file sp4c00224_si_001.pdf]

Supplementary Information for

Iodine activation from iodate reduction in aqueous films via photocatalyzed and dark reactions

Mago Reza,[ab] Lucia Iezzi,[c] Henning Finkenzeller,[ab] Antoine Roose,[c] Markus Ammann,[c] and Rainer Volkamer[ab*]

[a] *Department of Chemistry, University of Colorado Boulder, 80309 Boulder, CO, USA*
[b] *Cooperative Institute for Research in Environmental Sciences (CIRES), University of Colorado Boulder, 80309 Boulder, CO, USA*
[c] *Laboratory of Atmospheric Chemistry, Paul Scherrer Institute, 5232 Villigen, Switzerland*

[*]Rainer Volkamer
Email: rainer.volkamer@colorado.edu

This PDF file includes:

Supplementary Information Text
Figure S1
Tables S1 to S3
SI References

Supplementary Information Text

Expected concentration of iodate in the upper troposphere-lower stratosphere (UTLS)

According to Table 1 in Koenig et al. 2020[1], iodide (I^-) comprises 44% of particulate iodine in the UTLS, with a reported molality of 59 mmol/kg. Considering particulate iodine exists as iodide (I^-) and iodate (IO_3^-), gives a complimentary particulate iodate abundance, IO_3^-/Iy_{part}, of 56%. Using the proportional relationship of IO_3^-/ I^- abundance (and concentration), and solving for the molality of iodate gives $[IO_3^-]$ = 75.1 mmol/kg

$$\frac{IO_3^-/Iy_{part}}{I^-/Iy_{part}} = \frac{[IO_3^-], \mathrm{mmol/kg}}{[I^-], \mathrm{mmol/kg}}$$

Given that tropospheric and stratospheric aerosol tend to be acidic, the density of water and sulfuric acid, ρH_2O ~1.0 g/mL (1 kg/L); ρH_2SO_4 ~1.8 g/mL (1.8 kg/L), is used to estimate the range of the molarity of iodate in aerosols in the UTLS.

Molarity (mmol/L) = molality (mmol/kg) · density (kg/L)

Considering the approximated densities above results in the following range of $[IO_3^-]$ = 75 mmol/L - 135 mmol/L. While this range is not representative of the actual iodate concentrations in the UTLS, it serves as a lower and upper limit for them.

Expected concentration of H_2O_2 in the UT

Xuan et al., 2020[2] reports an H_2O_2 concentration in the aerosol phase of 0.093 ng/µg.

Given that tropospheric and stratospheric aerosol tend to be acidic, the density of water and sulfuric acid, ρH_2O ~1.0 g/mL (1 kg/L); ρH_2SO_4 ~1.8 g/mL (1.8 kg/L), is used to estimate the range of the molarity of H_2O_2 in aerosols in the UT: 0.0027 M – 0.005 M.

Molarity = mol H_2O_2 / L

mol H_2O_2 = 0.093×10^{-9} g / 34.0147 g/mol = 2.73×10^{-12} mol H_2O_2

L = 1×10^{-6} g of solvent (H_2O of H_2SO_4) / density (g/mL) /10^3

Concentration of H_2O_2 in the gas flow and in the film

The concentration of H_2O_2 expected in the gas flow was calculated using the Henry's law coefficient reported by O'Sullivan et al., 1996[3], approximated by considering the temperature of the bubbler at 18 °C, and the concentration of the H_2O_2 solution in the bubbler, e.g. [aq] = 0.098 M.

(1) K_H = e ^ [(A / T) – B]   (A = 7379, B =13.42)[3]

K_H = e ^ [(A / 291.15 K) – B]

$K_H = 1.51\times10^5$ M/atm

(2) K_H = [aq] / [gas]

[gas] = 6.49×10^{-7} atm

The concentration of H_2O_2 in the gas flow was then used to calculate the estimated concentration of H_2O_2 in the film, considering the temperature of the jacket at 20 °C

$K_H = e$ ^ [(A / 293.15 K) – B]

$K_H = 1.27x10^5$ M/atm

[aq] = 0.082 M

Characterization of background for dark H_2O_2 experiments

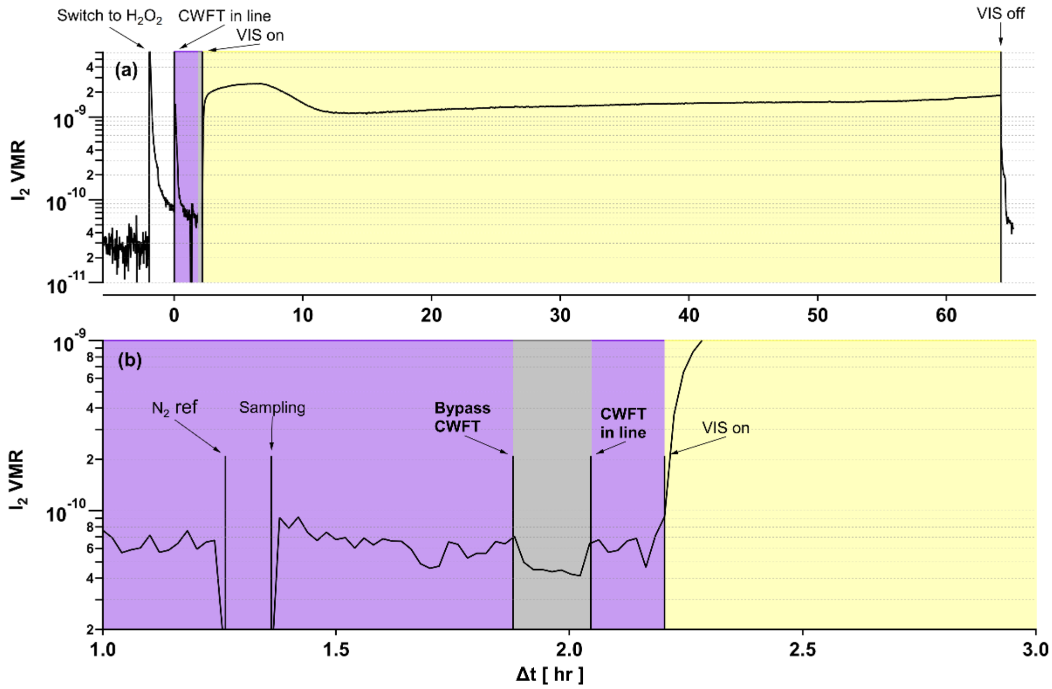

Figure S1 – Time-series of I_2 VMR for a dark-aging experiment of an iodate/CA matrix with H_2O_2 (5-minute running averaged data). a) The purple shaded region is when the film is exposed to H_2O_2 in the dark, this is followed by a period of irradiation with visible light, shaded in yellow. The first two observed peaks in I_2 are due to line conditioning; I_2 becomes mobilized with humidified carrier gas. b) The characterization of background I_2 for a dark experiment was done when the CWFT was bypassed and the carrier gas flowed through the H_2O_2 bubbler; the average I_2 detected while sampling the bypass line (shown as the grey line) is subtracted from the I_2 signal to give background corrected I_2 or delta ΔI_2.

Calculating Volume of Film

To estimate the volume of a film composed of sodium iodate, $NaIO_3$/ citric acid, CA/ Iron (III) citrate, Fe-Cit in the coated wall flow tube (CWFT) during an experiment, we take the definition of density, solving for volume of the film (V_{film}) gives:

(1) $$V_{film} = \frac{m_{film}}{\rho_{film}}$$

Where m_{film} is the total mass of the film and ρ_{film} is the density of the film. The total mass of the film is equal to the sum of its components:

(2) $$m_{film} = m_{NaIO_3} + m_{CA} + m_{Fe-Cit} + m_{H_2O}$$

The mass of the solutes is calculated from the initial concentration and volume of solution pipetted into the flow tube. The mass of the solvent, m_{H_2O} is the only unknown parameter. The water content in the

film depends on the hygroscopic properties of the film components and the relative humidity RH (%) that the film is exposed to during the experiment by the humidified gas flow going through the tube. Below, we detail the definitions and reasoning that serve to parameterize the microphysics of our system.

We start by considering that the ρ_{film} is dominated by the solutes' density, and can be calculated as a weighted mean of the density of the solutes:

(3) $$\rho_{\mathrm{film}} = \frac{n_{\mathrm{CA}} \cdot \rho_{\mathrm{CA}} + n_{\mathrm{Fe-Cit}} \cdot \rho_{\mathrm{Fe-Cit}} + n_{\mathrm{NaIO_3}} \cdot \rho_{\mathrm{NaIO_3}}}{n_{\mathrm{Total}}}$$

The density of a pure citric acid solution, ρ_{CA}, has been determined by Lienhard et al., 2012[4]. It depends on the mass fraction of CA, $\mathrm{mfs_{CA}}$, which in turn depends on the water activity (RH%), it is parameterized by the following equation (equation 7 from Lienhard et al., 2012[4]):

(4) $$\rho_{\mathrm{CA}}(\mathrm{mfs_{CA}}) = 0.9971 + d_1 \cdot \mathrm{mfs_{CA}} + d_2 \cdot \mathrm{mfs^2}_{\mathrm{CA}}$$

Where d_1 and d_2 are fit parameters from Table 1 in Lienhard et al., 2012[4]. The water activity of a pure CA solution as a function of mfs is given by the following empirical expression (equation 4 from Lienhard et al., 2012[4]):

(5) $$a_w\,(\mathrm{mfs_{CA}}) = \frac{1-\mathrm{mfs_{CA}}}{1+ q \cdot \mathrm{mfs_{CA}} + r \cdot \mathrm{mfs^2}_{\mathrm{CA}}}$$

The $\mathrm{mfs_{CA}}$ is solved for the water activity in our CWFT (RH%) from equation 5, with q and r representing the temperature and composition dependence of a_w for a given aqueous system:

(6) $$q = a_1 + a_2 \cdot T + a_3 \cdot T^2$$

and

(7) $$r = a_4 + a_5 \cdot T + a_6 \cdot T^2$$

The parameters a_1- a_6 have been determined for CA, and listed in Table 2 in Lienhard et al., 2012[4].

The density of an Fe-Cit solution, $\rho_{\mathrm{Fe-Cit}}$, is considered to be proportional to the density of a CA solution, ρ_{CA}, by a factor that is equal to the ratio between the densities of the pure solids, $\mathring{\rho}$.[5]

We treat the density contribution of a sodium iodate, $NaIO_3$ solution, $\rho_{\mathrm{NaIO_3}}$, as negligible. So, equation 3 becomes:

(8) $$\rho_{\mathrm{film}} = \frac{n_{\mathrm{CA}} \cdot \rho_{\mathrm{CA}} + n_{\mathrm{Fe-Cit}} \cdot \rho_{\mathrm{CA}} \cdot \frac{\mathring{\rho}_{\mathrm{CA}}}{\mathring{\rho}_{\mathrm{Fe-Cit}}}}{n_{\mathrm{CA}} + n_{\mathrm{Fe-Cit}}}$$

To calculate the m_{film}, we consider the mass fraction of solutes, mfs, which by definition, is equal to the ratio between the mass of the solutes and the total mass of the solution:

(9) $$\text{mfs} = \frac{m_{CA} + m_{Fe-Cit}}{m_{film}} = \frac{m_{CA} + m_{Fe-Cit}}{m_{CA} + m_{Fe-Cit} + m_{H_2O}}$$

To calculate the mass of H_2O, m_{H_2O}, we consider our citric acid / iron (III) citrate solution as a "pure citric acid solution" for simplicity. Our film has a certain water activity, a_w, that defines mfs_{CA} and the mass of our "pure citric acid solution", m_{CA}.

(10) $$mfs_{CA} = \frac{m_{CA}}{m_{CA} + m_{H_2O}}$$

Solving for m_{H_2O}, we obtain:

(11) $$m_{H_2O} = m_{CA} \cdot \frac{1 - mfs_{CA}}{mfs_{CA}}$$

To solve for m_{CA}, we first calculate the moles of our "pure citric acid solution", N_{cit}, considering the measured water activity of a 1 M iron (III) citrate solution was found to correspond to that of a 0.81 M aqueous CA solution.[5] Thus, we sum the actual moles of CA, n_{CA}, and the moles of iron(III) citrate, n_{Fe-Cit}, multiplied by 0.81 to account for the water activity correction:

(12) $$N_{cit} = n_{CA} + 0.81 \cdot n_{Fe-Cit}$$

Multiplying N_{cit} by the molar mass of CA we solve for the mass of a "pure citric acid solution", m_{CA}. So equation 11 becomes:

(13) $$m_{H_2O} = (N_{cit} \cdot MW_{CA}) \cdot \frac{1 - mfs_{CA}}{mfs_{CA}}$$

We plug in mfs_{CA} from the solution to equation 5 to calculate m_{H_2O}. We then calculate mfs and m_{film} by equation 9. Equation 8 gives us ρ_{film}, so now we can solve equation 1 and calculate the film volume for the matrix.

Example Film Volume Calculation for an iodate/CA film

Step 1 calculate the mass of CA used to coat the tube, m_{CA}**:**

Mass of CA used to make solution = 0.1927 g, Volume of solution = 10.00 mL, Molarity of solution = 0.1003 M

Volume of solution used to coat the tube = 0.0008 L

MW_{CA} = 192.124 g/mol

m_{CA} = Molarity of solution · 0.0008 L · MW_{CA}

m_{CA} = 0.01542 g

Step 2 calculate mfs_{CA} for the water activity of the film (RH of the carrier flow through the CWFT):

RH = 88.27%; use aw = 0.8827 to solve for mfs_{CA} in Equation 5.

$mfs_{CA} = 0.480$

Step 3 calculate the density of the film using CA as the matrix and treating the density contribution of $NaIO_3$ as negligible. Substituting $mfs_{CA} = 0.480$ into Equation 4:

$$\rho_{CA}(mfs_{CA}) = 1.2286\ g/cm^3$$

$$\rho_{film} = 1.2286\ g/cm^3$$

Step 4 calculate the m_{H_2O} of the film**,** substituting for mfs_{CA} and m_{CA} in Equation 11.

$$m_{H_2O} = 0.01668\ g$$

Step 5 calculate the mass of the film:

$$m_{film} = m_{CA} + m_{H_2O} = 0.01542\ g + 0.01668\ g$$

$$m_{film} = 0.0321\ g$$

Step 6: calculate the volume of the film by Equation 1:

$$V_{film} = \frac{m_{film}}{\rho_{film}} = \frac{0.0321\ g}{1.23\ g/cm^3} = 0.0261\ cm^3$$

$$V_{film} = 26.1\ mm^3$$

CE-DOAS fit settings

Table S1 – CE-DOAS fit settings used to retrieve I_2 slant column densities (SCDs).

Primary Species	I_2
Fitting window	508 – 558 nm
Cross-sections:	
I_2 (298 K)	Spietz et al. 2006[6]
H_2O	HITEMP (Rothman et al. 2010[7])
NO_2 (298 K)	Vandaele et al. 1998[8]
O_4 (293 K)	Thalman and Volkamer 2013[9]
Polynomial degree	Order 5 (six coefficients)

Comparison of theoretical and observed kinetics

Table S2 – kinetics comparison for the iodate reduction by H_2O_2 reaction, (part of the Bray-Liebhafsky mechanism) according to Schmitz and Furrow 2012[10].

H_2O_2: iodate	Matrix	$[H_2O_2]$ (M)	$[IO_3^-]$ (M)	$[H^+]$ (M)	k_a ($M^{-1}s^{-1}$)	k_b (s^{-1})	Theo. prod rate I_2 (Ms^{-1})	Avg. exp. prod rate I_2 (Ms^{-1})	Factor (Theo/ Measured)	L (cm)
2.1:1	CA	0.082	0.034	0.042	7.53E-7	2.56E-8	1.05E-09	3.22E-10	3.3	28
2.3:1	CA	0.082	0.031	0.042	7.59E-7	2.38E-8	9.74E-10	5.10E-10	1.9	29
2.7:1	CA	0.082	0.026	0.043	7.70E-7	2.00E-8	8.21E-10	3.58E-10	2.3	32
1:1.8	CA	0.082	0.135	0.031	6.02E-7	8.12E-8	3.33E-09	2.16E-10	15.4	16
2.8:1	CA	0.421	0.135	0.031	6.02E-7	8.12E-8	*1.71E-08	2.90E-09	5.9	16
14:1	CA	0.421	0.026	0.043	7.70E-7	2.00E-8	*4.22E-09	4.27E-09	1.0	32
2.5:1	HSO_4^-	0.082	0.021	0.159	2.52E-6	5.27E-8	2.16E-09	5.42E-10	4.0	19
2.7:1	HSO_4^-	0.082	0.019	0.160	2.53E-6	4.83E-8	1.98E-09	7.21E-10	2.8	20
2.8:1	HSO_4^-	0.082	0.018	0.161	2.54E-6	4.62E-8	1.89E-09	7.26E-10	2.6	21
3.0:1	BTCA	0.082	0.025	0.028	5.53E-7	1.37E-8	5.60E-10	1.03E-09	0.6	38
3.2:1	BTCA	0.082	0.024	0.028	5.55E-7	1.31E-8	5.37E-10	1.57E-09	0.3	39

*Theoretical production rates of I_2 (Ms^{-1}) that are not expected to follow the rate law in Schmitz and Furrow 2012[10] due to high $[H_2O_2]$ (> 0.1 M).

Calculating theoretical production rate of I_2

The concentrations of hydronium $[H^+]$ and iodate $[IO_3^-]$ in the film were calculated accounting for the formation/dissociation of iodic acid,[11] using the acid dissociation constant, K_a, of the acid making up the matrix (either CA, ABS, or BTCA).

The approximation of the reaction rate coefficient for the reaction between H_2O_2 and iodate (in the bulk), k_a ($M^{-1}s^{-1}$), was calculated using the equation from Schmitz and Furrow 2012[10]:

(1) $$k_a = K' + K'' \cdot [H^+]$$

where K' = 1.3×10^{-7} $M^{-1}s^{-1}$ (20°C) and K" = 1.5×10^{-5} $M^{-2}s^{-1}$ (25°C)

The rate law equation gives the theoretical consumption rate of iodate, L_{IO3-}, ($M\ s^{-1}$):

(2) $$L_{IO3-} = k_a \cdot [IO_3^-] \cdot [H_2O_2]$$

The theoretical production rate of iodine, P_{I2} ($M\ s^{-1}$) was calculated by multiplying the approximated reaction rate coefficient by the concentration of iodate and H_2O_2 in the film and by the molar ratio between I_2 and iodate:

(3) $$P_{I2} = \frac{1}{2} \cdot L_{IO3-} = \frac{1}{2} k_a \cdot [IO_3^-] \cdot [H_2O_2]$$

The reacto-diffusive length, L (cm), was calculated as follows:

(4) $$L = \sqrt{\frac{D}{k_b}}$$

Where D (the diffusivity coefficient of H_2O in a Fe-Cit/CA solution) is approximated to $7x10^{-6}$ cm^2 s^{-1} [12,13] and k_b is the pseudo first order rate coefficient, (s^{-1}), for the bulk reaction between H_2O_2 and iodate, calculated as follows:

(5) $$k_b = k_a \cdot [IO_3^-]$$

Table S3 – Comparison of percent iodate consumed with changing molar ratio (H_2O_2:iodate) for the iodate/CA matrix

Molar Ratio<br>H_2O_2:Iodate	Light	H_2O_2 in CWFT<br>ppmv [M]	Time (h)	(%) Iodate consumed	Factor<br>(Theo/<br>Measured)
2.1:1	Dark	0.65 [0.082]	0.57<br>16.32	0.0036<br>0.035	3.3
	VIS	0.65 [0.082]	1.40<br>3.41	4.3<br>13	
2.3:1	Dark	0.65 [0.082]	0.58	0.13	1.9
	VIS	0.65 [0.082]	1.46<br>62.02	2.1<br>64	
2.7:1	Dark	0.65 [0.082]	0.54<br>16.35	0.19<br>0.48	2.3
	VIS	0.65 [0.082]	1.45	2.3	
1:1.8	Dark	0.65 [0.082]	15.27	0.018	15.4
2.8:1	Dark	3.3 [0.421]	16.00<br>67.17	0.43<br>37	5.9
14:1	Dark	3.3 [0.421]	16.01<br>18.55	2.45<br>3.1	1.0

References

(1) Koenig, T. K.; Baidar, S.; Campuzano-Jost, P.; Cuevas, C. A.; Dix, B.; Fernandez, R. P.; Guo, H.; Hall, S. R.; Kinnison, D.; Nault, B. A.; Ullmann, K.; Jimenez, J. L.; Saiz-Lopez, A.; Volkamer, R. Quantitative Detection of Iodine in the Stratosphere. *Proc. Natl. Acad. Sci.* **2020**, *117* (4), 1860–1866. https://doi.org/10.1073/pnas.1916828117.

(2) Xuan, X.; Chen, Z.; Gong, Y.; Shen, H.; Chen, S. Partitioning of Hydrogen Peroxide in Gas-Liquid and Gas-Aerosol Phases. *Atmospheric Chem. Phys.* **2020**, *20* (9), 5513–5526. https://doi.org/10.5194/acp-20-5513-2020.

(3) O'Sullivan, D. W.; Lee, M.; Noone, B. C.; Heikes, B. G. Henry's Law Constant Determinations for Hydrogen Peroxide, Methyl Hydroperoxide, Hydroxymethyl Hydroperoxide, Ethyl Hydroperoxide, and Peroxyacetic Acid. *J. Phys. Chem.* **1996**, *100* (8), 3241–3247. https://doi.org/10.1021/jp951168n.

(4) Lienhard, D. M.; Bones, D. L.; Zuend, A.; Krieger, U. K.; Reid, J. P.; Peter, T. Measurements of Thermodynamic and Optical Properties of Selected Aqueous Organic and Organic–Inorganic Mixtures of Atmospheric Relevance. *J. Phys. Chem. A* **2012**, *116* (40), 9954–9968. https://doi.org/10.1021/jp3055872.

(5) Dou, J.; Alpert, P. A.; Corral Arroyo, P.; Luo, B.; Schneider, F.; Xto, J.; Huthwelker, T.; Borca, C. N.; Henzler, K. D.; Raabe, J.; Watts, B.; Herrmann, H.; Peter, T.; Ammann, M.; Krieger, U. K. Photochemical Degradation of Iron(III) Citrate/Citric Acid Aerosol Quantified with the Combination of Three Complementary Experimental Techniques and a Kinetic Process Model. *Atmospheric Chem. Phys.* **2021**, *21* (1), 315–338. https://doi.org/10.5194/acp-21-315-2021.

(6) Spietz, P.; Gómez Martín, J.; Burrows, J. P. Effects of Column Density on I2 Spectroscopy and a Determination of I2 Absorption Cross Section at 500 Nm. *Atmospheric Chem. Phys.* **2006**, *6* (8), 2177–2191. https://doi.org/10.5194/acp-6-2177-2006.

(7) Rothman, L. S.; Gordon, I. E.; Barber, R. J.; Dothe, H.; Gamache, R. R.; Goldman, A.; Perevalov, V. I.; Tashkun, S. A.; Tennyson, J. HITEMP, the High-Temperature Molecular Spectroscopic Database. *J. Quant. Spectrosc. Radiat. Transf.* **2010**, *111* (15), 2139–2150. https://doi.org/10.1016/j.jqsrt.2010.05.001.

(8) Vandaele, A. C.; Hermans, C.; Simon, P. C.; Carleer, M.; Colin, R.; Fally, S.; Mérienne, M. F.; Jenouvrier, A.; Coquart, B. Measurements of the NO2 Absorption Cross-Section from 42 000 Cm−1 to 10 000 Cm−1 (238–1000 Nm) at 220 K and 294 K. *J. Quant. Spectrosc. Radiat. Transf.* **1998**, *59* (3–5), 171–184. https://doi.org/10.1016/S0022-4073(97)00168-4.

(9) Thalman, R.; Volkamer, R. Temperature Dependent Absorption Cross-Sections of O2–O2 Collision Pairs between 340 and 630 Nm and at Atmospherically Relevant Pressure. *Phys. Chem. Chem. Phys.* **2013**, *15* (37), 15371. https://doi.org/10.1039/c3cp50968k.

(10) Schmitz, G.; Furrow, S. Kinetics of the Iodate Reduction by Hydrogen Peroxide and Relation with the Briggs–Rauscher and Bray–Liebhafsky Oscillating Reactions. *Phys. Chem. Chem. Phys.* **2012**, *14* (16), 5711. https://doi.org/10.1039/c2cp23805e.

(11) Schmitz, G. Iodine Oxidation by Hydrogen Peroxide in Acidic Solutions, Bray-Liebhafsky Reaction and Other Related Reactions. *Phys. Chem. Chem. Phys.* **2010**, *12* (25), 6605. https://doi.org/10.1039/b927432d.

(12) Dou, J.; Luo, B.; Peter, T.; Alpert, P. A.; Corral Arroyo, P.; Ammann, M.; Krieger, U. K. Carbon Dioxide Diffusivity in Single, Levitated Organic Aerosol Particles. *J. Phys. Chem. Lett.* **2019**, *10* (15), 4484–4489. https://doi.org/10.1021/acs.jpclett.9b01389.

(13) Lienhard, D. M.; Huisman, A. J.; Bones, D. L.; Te, Y.-F.; Luo, B. P.; Krieger, U. K.; Reid, J. P. Retrieving the Translational Diffusion Coefficient of Water from Experiments on Single Levitated Aerosol Droplets. *Phys. Chem. Chem. Phys.* **2014**, *16* (31), 16677. https://doi.org/10.1039/C4CP01939C.
